# Ikigai as a Personal Resource for Work Engagement: A Cross-Sectional Study Among Nursing Trainees in Germany

**DOI:** 10.3390/nursrep15070225

**Published:** 2025-06-24

**Authors:** Clemens Koob, Claudine M. Tomic

**Affiliations:** Department of Health and Nursing, Catholic University of Applied Sciences Munich, Preysingstraße 95, 81667 Munich, Germany

**Keywords:** work engagement, ikigai, personal resources, job demands–resources theory, nursing trainees, nursing management, nursing education, self concept, Germany

## Abstract

**Background/Objectives:** Work engagement is essential for quality care and workforce retention in professional nursing. While job demands–resources theory has guided extensive research on job-related antecedents, personal resources have received comparatively less empirical attention, primarily focusing on self-efficacy, self-esteem, and optimism. This study examined the unique association between ikigai—the Japanese concept of life purpose—as a novel personal resource and work engagement in professional nursing practice, controlling for established job resources, demands, and personal resources. **Methods**: An analytical cross-sectional study was conducted with vocational nursing trainees in Germany (N = 166). Data were collected via online questionnaire using validated instruments to assess ikigai, job resources (autonomy, interpersonal relations, professional resources), job demands (work overload, lack of formal rewards), other personal resources (self-efficacy, organization-based self-esteem, optimism), and work engagement. Hierarchical multiple linear regression examined ikigai’s unique association with work engagement. **Results**: The final model explained 40.3% of variance in engagement, with ikigai accounting for a statistically significant increase in explained variance (ΔR^2^ = 0.033, *p* < 0.01). Ikigai demonstrated a unique positive association with work engagement (β = 0.24, *p* < 0.01), comparable in strength to job resources and other personal resources. **Conclusions**: Findings support ikigai as a distinct personal resource associated with work engagement among nursing trainees. This extends the job demands–resources model by highlighting the relevance of existential constructs. Supporting ikigai development may offer a complementary strategy for promoting engagement in professional nursing.

## 1. Introduction

Work engagement of nursing professionals is considered pivotal in research [1,2,3,4,5]. It denotes a positive, fulfilling, work-related psychological state characterized by vigor, dedication, and absorption [6]. Vigor reflects high energy, effort, and persistence even in the face of difficulties. Dedication refers to strong involvement, experience of meaning, and enthusiasm [6,7]. Absorption implies being fully focused on and immersed in one’s work [6,7]. Together, these dimensions reflect elevated energy and strong identification with one’s work. Prior research has consistently linked work engagement to a range of positive outcomes, including greater wellbeing and job satisfaction [1,6,8], enhanced performance and care (e.g., higher task performance and work effectiveness) [1,6,8], extra-role behaviors (e.g., Organizational Citizenship) [6,9], stronger job commitment, lower turnover [1,6,8], and reduced intent to leave the nursing profession [1].

From a nursing management perspective, this raises the question of which factors shape work engagement in nursing practice. Job demands–resources theory is commonly employed to examine such antecedents [6,7]. This theory posits that every job involves demands and resources, excessive demands diminish engagement, and resources—defined as physical, psychological, social, and organizational aspects that facilitate attaining work-related goals—enhance it [6]. Numerous studies have investigated these antecedents of work engagement in general [8,10] and specifically in nursing [1,10]. More recently, research has focused on the key job resources and demands delineated as particularly relevant to nursing [11]. Within this framework, Bartsch et al. [2] found that autonomy, professional resources, and interpersonal relationships were positively associated with nurses’ engagement, while lack of formal rewards had a negative association and work overload had a positive association. Building on the same framework, Forster and Koob [4] found that empowering leadership was positively related to engagement among nurse managers, while lack of formal rewards and work–life interferences were negatively associated with engagement.

A further proposition of the job demands–resources theory is that, in addition to job resources, personal resources are also capable of evoking work engagement [6]. Personal resources denote individual-level factors in terms of positive beliefs about one’s self (e.g., self-esteem) and the world (e.g., optimism) [12]. Personal resources are self-beliefs of resiliency, or positive self-evaluations that refer to one’s sense of the ability to control and impact the environment successfully [6,13]. In contrast to the plethora of studies on job resources and demands, comparatively fewer studies have focused on the contribution of personal resources to work engagement, both in general [6,8] and in the specific context of nursing [1]. Yet, a recent meta-analysis by Mazzetti et al. [8] suggests that the impact of personal resources may even surpass that of job resources. Extant research in the personal resources domain has primarily examined self-efficacy [8,12,13,14], optimism [8,12,13,14], and self-esteem [12,13], all of which have demonstrated positive associations with work engagement. Given the theoretical and practical importance of work engagement, identifying additional personal resources that may evoke engagement remains a valuable research objective.

Poindexter [15] and Kotera et al. [16] have recently proposed that *ikigai* could represent a promising additional personal resource. The Japanese concept of ikigai—with “iki” meaning “to live” and “gai” meaning “reason”—refers to a sense of life purpose or a reason for living [17,18]. Although the concept and its structure continue to be debated [19], ikigai is commonly considered a composite construct comprising meaning, motivations, and values in life [20,21,22]. Kumano [21] delineated life-affirmation, goals and dreams, sense of value, meaning of life, a sense of fulfilment, and commitment as six key components of ikigai. A three-dimensional model of ikigai that informs current research conceptualizes it as encompassing (1) positive emotions towards life, (2) active and positive attitudes towards one’s future, and (3) acknowledgment of the meaning of one’s existence [23,24,25]. Aside from studies pertaining to the translation and cross-cultural validation of instruments measuring ikigai [24,25,26], empirical research on ikigai has primarily centered on health-related outcomes, where it is considered a predictor of physical, psychological, and social health [27]. For instance, higher levels of ikigai have been associated with lower risk of developing functional disability [28,29], frailty [30], dementia, hopelessness, and depressive symptoms, and with greater happiness and life satisfaction [29] and lower risk of suicidal ideation [31]. Regarding health-related behaviors, greater ikigai has been linked to less problematic smartphone use [32] and higher likelihood of utilizing preventive health services [33]. Notably, a lacking sense of ikigai has been associated with increased all-cause mortality risk [34]. Despite this growing body of evidence regarding its health implications, the potential relationship between ikigai and motivational workplace outcomes—such as work engagement—remains underexplored in empirical research, even though conceptual commentaries have highlighted its potential relevance.

This study addresses this gap. Specifically, the aim is to examine ikigai as a personal resource related to work engagement in professional nursing. According to the direct effects model [12], we hypothesize that ikigai has a direct and positive association with work engagement, independent of job resources, job demands, and other personal resources. Two theoretical frameworks support this hypothesis.

First, the broaden-and-build theory posits that positive emotions expand an individual’s cognitive and behavioral repertoire in terms of broadening the scope of attention, cognition, and action [35]. Over time, frequent positive emotional experiences at work may translate into a sustained motivational state of energy, dedication, and immersion in work, i.e., work engagement [36]. Since ikigai essentially involves positive emotions towards life, it can be assumed that these positive emotions also materialize in work life and then translate into work engagement, according to the broaden-and-build theory.

Second, the theory of reasoned goal pursuit [37] suggests that an individual’s motivation to engage stems from aspiration to attain certain goals. A key component is “procurement goals”, the experiences and outcomes one prefers to achieve [37]. Because ikigai implies having a life purpose and active and positive attitudes towards one’s future, it may act as a source of intrinsically desirable procurement goals in work life, providing a motivational foundation for goal-directed behavior at work. When work tasks are experienced as instrumental to the realization of ikigai-related goals, individuals may be more likely to exhibit higher levels of work engagement.

Beyond offering a theoretical rationale for ikigai’s motivational function, this framework also supports its conceptual distinctiveness as a personal resource. While constructs such as self-efficacy, self-esteem, and optimism focus on confidence in handling tasks, self-worth, or positive expectations, ikigai reflects a broader existential orientation, combining emotional positivity with long-term purpose and a sense of societal contribution. Unlike narrower self-evaluative concepts or a general future-oriented outlook, ikigai connects life meaning with enduring aspirations, offering a purpose-driven motivational base. Thus, we theorize that ikigai may exert a direct, positive effect on work engagement by energizing behavior around intrinsically valued procurement goals.

Rather than examining ikigai in isolation, this study incorporates it alongside other job resources, job demands, and personal resources, to capture the unique association of ikigai with work engagement when controlling for other factors. This multivariable approach is consistent with the job demands–resources model’s principle that these domains should be assessed jointly [6].

Furthermore, our focus on vocational nursing trainees in Germany provides an important empirical context. Nursing trainees play an important role in care delivery by assisting with fundamental tasks under supervision or even more autonomously performing patient care collaboratively in interprofessional teams [38]. More importantly, they represent a critical component of the future nursing workforce and thus are central to mitigate staff shortages [39,40,41,42]. Prior research highlights five core developmental challenges in vocational nursing training, ranging from building nursing competence and shaping nursing relationships to coping with existential experiences, and from developing an understanding of oneself to integrating into healthcare organizations [43,44]. Engagement during training is pivotal for mastering these developmental processes.

Beyond contributing to the literature, this study seeks to inform nursing education and management by providing novel directions for strengthening work engagement.

In summary, this study investigates whether ikigai functions as a personal resource that is uniquely associated with work engagement in vocational nursing trainees. We aimed to test this relationship while accounting for established job resources, job demands, and other personal resources.

## 2. Materials and Methods

### 2.1. Study Design

To investigate the association between ikigai and nursing trainees’ work engagement, an analytical, cross-sectional study design was employed. As the study examined associations between variables without experimental manipulation, it can also be characterized as correlational in nature. A structured online questionnaire was used to collect data, utilizing instruments that had demonstrated validity and reliability in prior studies. Unless otherwise noted, all instruments used in this study were freely available through their original publications. All items were presented in the German language. The survey was implemented using the SoSci Survey platform. Prior to fielding the survey, a pre-test was performed, and the instrument was refined based on participant feedback. Data collection took place in April and May 2024.

### 2.2. Participants

Individuals were eligible for study participation if they were pursuing vocational nursing training leading to qualification as a general nurse, in accordance with the German Act on the Nursing Professions (Pflegeberufegesetz—PflBG [45]), and if they were at least 16 years of age at the time of the survey. In Germany, vocational nursing training has a generalist structure, preparing trainees to deliver care in various settings, including acute and long-term inpatient care as well as outpatient services. Training typically lasts three years on a full-time basis and comprises both theoretical and practical instruction at nursing schools and practical training in facilities including hospitals, nursing homes, outpatient services, and psychiatric and pediatric care facilities. Trainees from all years of vocational training were eligible to participate in this study.

Individuals enrolled in university-based nursing programs under the same legislative framework were excluded. Likewise, state-certified nursing professionals already holding the titles of “general nurse”, “healthcare worker and nurse”, “healthcare and pediatric nurse”, or “geriatric nurse” were not eligible. This exclusion also applied to nursing assistants, nursing specialist assistants, and those in managerial roles.

The study employed a convenience sampling approach. Participants were recruited through a structured outreach to nursing training schools. Given the large number of such institutions in Germany (over 1300 [46]), sampling was geographically limited to 146 nursing training schools located in the federal state of Bavaria (as of April 2024), for reasons of research feasibility. Initial contact was made via telephone, and schools that generally agreed to participate were subsequently sent an email containing the survey link and an informational flyer. Schools were invited to disseminate these materials to eligible trainees either via circular e-mail from administration or by instructors during class sessions.

### 2.3. Measures

#### 2.3.1. Work Engagement

Work engagement was assessed using the 3-item ultra-short version of the Utrecht Work Engagement Scale (UWES-3) [47]. The UWES is the most widely used measure of work engagement in academic research [8], and the UWES-3 has demonstrated favorable psychometric properties across diverse international populations. In large national datasets from Finland, Japan, the Netherlands, Belgium, and Spain, comprising various occupational groups including health professionals, the UWES-3 showed adequate internal consistency (Cronbach’s alpha 0.77–0.85), high shared variance with UWES-9 (0.86–0.92), validity through comparable associations with engagement antecedents and outcomes, as well as discriminant validity against other work-related wellbeing measures [47]. The UWES-3 has also been applied in the German-language version of the European Working Conditions Survey, targeting employed residents aged 16 and older across 35 countries, where it demonstrated acceptable reliability in all countries, including Germany [48]. In addition, a student-adapted German version was validated among students from various programs, including health-related fields [49], supporting its use in educational contexts.

The three items were rated on a 7-point Likert scale ranging from “never” to “always,” with higher scores indicating stronger engagement. An example item is: “I am enthusiastic about my job.” Participants responded in reference to their current practical training placements.

#### 2.3.2. Ikigai

The German version of the Ikigai-9 scale (Ikigai-9-G) was used to measure trainees’ sense of purpose in life. Originally developed in Japan [23], the scale was recently validated in a large, nationally representative quota sample of German adults (N = 5000) [24]. The validation study demonstrated sound psychometric properties, including high internal consistency (Cronbach’s alpha = 0.88; McDonald’s omega = 0.88), structural validity, and concurrent validity through associations with life satisfaction, health-related quality of life, and depressive symptoms [24].

The scale comprises nine items covering three dimensions: (1) positive emotions regarding life (e.g., “I often feel that I am happy”), (2) active and positive attitudes toward one’s future (e.g., “I would like to develop myself”), and (3) recognition of the meaning of one’s existence (e.g., “I feel that I am contributing to someone or society”). Items were rated on a 5-point Likert scale from “does not apply” to “applies a lot,” with higher scores reflecting stronger ikigai.

#### 2.3.3. Personal Resources

Following Xanthopoulou et al. [13], personal resources (excluding ikigai) were operationalized as a composite of self-efficacy, organization-based self-esteem, and optimism.

Self-efficacy, referring to one’s beliefs about one’s capabilities to control events that affect one’s life, was captured using the 3-item General Self-Efficacy Short Scale (ASKU) [50], with items such as “I can cope well with most problems on my own.” The scale was validated in two quota samples of German-speaking adults and a representative sample of the German residential population. Across these samples, the scale demonstrated satisfactory internal consistency (McDonald’s omega 0.81–0.86), factorial validity, convergent validity, and criterion validity with respect to a broad range of sociodemographic and psychological variables [50]. Responses were given on a 5-point Likert scale from “does not apply at all” to “fully applies.”

Organization-based self-esteem, reflecting perceived personal value within the organizational context, was assessed using the validated German version of the 10-item organization-based self-esteem scale (OBSE) [51,52] (e.g., “I am valuable”). The scale was validated in three independent samples of German nurses and nursing trainees. Across these samples, it demonstrated sound psychometric properties, including adequate internal consistency (Cronbach’s alpha 0.88–0.91), factorial validity, construct validity with respect to psychological variables and personality traits, and incremental validity in predicting job satisfaction and performance [51]. Items were rated on a 7-point scale from “does not apply at all” to “fully applies.” Respondents were instructed to refer their answers to their current training placement.

Optimism, i.e., the tendency to believe that one will generally experience good outcomes in life, was captured using the 3-item optimism subscale of the validated German version of the Revised Life Orientation Test (LOT-R) [53,54]. The scale was validated in a representative sample of 2372 adults in Germany and demonstrated satisfactory psychometric properties, including internal consistency (Cronbach’s alpha 0.70 for the optimism subscale) and structural validity. Evidence for convergent validity was found through associations with variables such as subjective health status [55]. An example item was “In uncertain times, I usually expect the best”. A 5-point Likert response scale was employed, ranging from “strongly disagree” to “strongly agree”.

To ensure comparability across scales, all 5-point responses were linearly transformed to a 7-point scale. A composite personal resources score was then calculated as the mean of the three subscales, with higher scores indicating stronger personal resources.

#### 2.3.4. Job Resources

Job resources were conceptualized as a composite of autonomy, interpersonal relations, and professional resources, identified as key dimensions of nursing-related job resources in an integrative review by Broetje et al. [11] and shown to be associated with work engagement among German nursing professionals [2]. All constructs were measured using the validated ReA questionnaire [56], which was developed and tested in two samples of German-speaking adults employed in full- and part-time positions across different occupational sectors (N = 1600). Permission to use the ReA questionnaire was obtained from its developers.

Autonomy in the current training placement was assessed with a 3-item autonomy subscale (e.g., “I can decide for myself when to complete tasks”). In the original validation study, this subscale demonstrated high internal consistency, with Cronbach’s alpha of 0.90 reported in both independent samples [56].

Interpersonal relations with peers, referring to supportive, respectful, and appreciative relationships with colleagues, were measured using the 3-item support from colleagues subscale (e.g., “I can ask my colleagues for support at any time”). In the validation study, this subscale demonstrated excellent internal consistency, with Cronbach’s alphas of 0.91 and 0.93 reported across the two independent samples [56].

Professional resources, denoting the organizational and physical aspects that support the provision of high-quality nursing care, were assessed using two 3-item subscales: role clarity (referring to work organization, e.g., “I always know who is responsible for what”) and straining work environment (referring to physical work environment aspects, e.g., “The ambient factors at my workplace impede my work”). In the original validation study, the role clarity subscale demonstrated Cronbach’s alphas of 0.64 and 0.73, while the straining work environment subscale showed higher internal consistency, with alphas of 0.88 and 0.91 [56]. Items from the straining work environment subscale were reverse coded prior to score aggregation so that higher scores indicated stronger resources.

All items were rated on a 6-point Likert scale from “does not apply at all” to “fully applies.” A composite score for job resources was calculated as the mean of the autonomy, interpersonal relations, and professional resources scales, with higher scores reflecting stronger job resources.

#### 2.3.5. Job Demands

Job demands were operationalized as a composite of work overload and lack of formal rewards, based on Broetje et al. [11] and prior findings from Bartsch et al. [2].

Work overload, referring in particular to the quantitative amount of work needing to be done within a certain amount of time [11], was assessed using the 3-item time pressure subscale from the ReA questionnaire [56] (e.g., “I am always under great time pressure”). In the original validation study, this subscale demonstrated good internal consistency, with Cronbach’s alphas of 0.82 and 0.84 [56].

To capture whether the nursing trainees perceived a lack of formal rewards with regard to the training allowance paid by their practical training provider, the 4-item pay subscale from the German version of the Job Satisfaction Survey (JSS-German) was used [57,58] (e.g., “I feel unappreciated by the organization when I think about what they pay me”). The items were rated on a 6-point Likert scale from “disagree very much” to “agree very much.” In the original validation study, the subscale demonstrated acceptable internal consistency with a Cronbach’s alpha of 0.75 [57]. Two items in the pay subscale were reverse coded prior to score aggregation so that higher scores consistently indicated a greater lack of formal rewards.

A total job demands score was computed as the mean of the two subscales, with higher scores reflecting stronger job demands.

#### 2.3.6. Baseline Data and Control Variables

In line with recommendations for focused use of control variables [59,60], participants’ age, gender, year of training, and current practical training setting were recorded both for description of the sample and as potential confounders.

### 2.4. Bias and Data Quality

To address concerns regarding common method bias, we followed procedural remedies recommended by Podsakoff et al. [61]. The questionnaire was structured into clearly separated sections with transitional instructions to help psychologically separate constructs. Response options were verbalized to ensure consistency of understanding. To minimize method bias from common scale properties, varied response formats and verbal anchors were employed, in alignment with the original instruments. Anonymity was guaranteed in order to reduce social desirability bias. The questionnaire was kept concise to minimize respondent fatigue and promote thoughtful responses.

To ensure data quality, screening questions were used to verify eligibility. Additionally, the longstring index—defined as the maximum number of identical consecutive responses—was calculated for two item sets: the Ikigai-9-G and a block of 19 consecutive items that were scored on a 6-point Likert scale. Participants with a longstring value exceeding 7 in either case were excluded, as such patterns are indicative of inattentive or patterned responding [62].

### 2.5. Study Size

The required sample size was determined via a priori power analyses conducted in G*Power (V3.1.9.6 for Mac).

First, the necessary sample size for detecting a statistically significant joint effect of the predictor variables on the outcome was determined. To assess the combined effect of all independent variables, Cohen’s f^2^ was used as the effect size measure, consistent with standard practice in multiple linear regression [63]. According to Cohen [63], f^2^ = 0.02 denotes a small effect, f^2^ = 0.15 a moderate effect, and f^2^ = 0.35 a large effect. Based on previous research on work engagement within the job demands–resources framework, e.g., [1,8], a medium to large joint effect was deemed plausible. Therefore, an expected effect size of f^2^ = 0.15 was set for the power analysis. With α = 0.05, f^2^ = 0.15, target power 1-ß = 0.80, and 11 predictor variables, the analysis indicated a required total sample size of 123 participants.

Second, to determine the sample size needed to detect the incremental contribution of ikigai beyond the baseline model, a separate a priori power analysis was conducted using a hierarchical multiple regression framework (fixed model, R^2^ increase) [64]. Cohen’s f^2^ was used again as the effect size measure for the proportion of additional variance explained by ikigai. As f^2^ is calculated as $f^{2}=∆R^{2}/(1-R_{Full\;Model}^{2})$, and given the absence of prior empirical studies on ikigai in this context, we conservatively assumed $∆R^{2}=0.03$ and a total R^2^ for the full model of 0.40, resulting in an estimated f^2^ = 0.05. With α = 0.05, f^2^ = 0.05, target power = 0.80, one tested predictor, and 11 predictors in total, the analysis indicated a required sample size of 160 participants.

Based on these analyses, we set the minimum required sample size for the study at 160 participants.

### 2.6. Statistical Analyses

All analyses were conducted using SPSS Statistics (V29), R (V4.3.2), and RStudio (V2023.06.1+524).

The dataset was initially cleaned by removing cases that did not meet the inclusion criteria. Missing data were assessed for adherence to the Missing Completely at Random (MCAR) assumption using Jamshidian-Jalal’s test from the MissMech package (V1.0.2). The results (Hawkins test *p* < 0.001; non-parametric test of homoscedasticity *p* = 0.69) did not provide sufficient evidence to reject the MCAR assumption. Under MCAR, listwise deletion does not bias results [65]; therefore, cases with missing data were excluded. Furthermore, we excluded cases that failed the longstring checks, with longstring indices computed using the careless package (V1.2.2).

Following this, the sample structure was described, and the means, standard deviations, correlations, Cronbach’s alphas, and McDonald’s omegas of the study variables were computed. McDonald’s omega was included as it provided a more general estimate of internal consistency under a less restrictive congeneric model that did not assume tau equivalence, as required by Cronbach’s alpha [66]. This is particularly important for short or potentially heterogeneous scales, where omega may more accurately reflect reliability [67].

To examine the unique association of ikigai with work engagement, hierarchical multiple linear regression was conducted. Prior to the main analysis, regression assumptions were tested using the performance and car packages. Linearity between the dependent variable and the predictor variables was assessed using residuals vs. fitted values plot and partial residual plots of the predictor variables, which showed only minor deviations from linear relations. Multicollinearity was not a concern, as the maximum variance inflation factor (VIF) was 1.80, well below the threshold of 10 [68]. Normality of residuals was supported by the histogram, Q-Q plot, and the Shapiro–Wilk test (*p* = 0.46). Visual inspection of the scale–location plot and the Breusch–Pagan test (*p* = 0.27) did not indicate heteroscedasticity. In the outlier analysis using Mahalanobis distance (threshold = 32.91) [69] and Cook’s distance (threshold = 0.95) [70,71], no participants were identified as outliers. Therefore, we proceeded with the main analysis.

In Model 1, job resources, job demands, personal resources (excluding ikigai), and control variables (age, gender, year of training, current training setting) were included as predictors. Categorical control variables were dummy-coded, with j–1 dummy variables used for each variable with j categories. In Model 2, ikigai was added to assess its incremental contribution to work engagement.

Cohen’s f^2^ was used to evaluate the strength of the joint association between predictors and work engagement, as well as the incremental variance explained by ikigai. Values of 0.02, 0.15, and 0.35 were interpreted as small, medium, and large effect sizes, respectively. Standardized regression coefficients (β) were used to assess the strength of individual predictors in the final model, with absolute values < 0.2 considered small, 0.2–0.5 moderate, and >0.5 strong. A *p*-value < 0.05 was considered statistically significant.

To further assess the discriminant validity of ikigai relative to the personal resources composite, we conducted a post hoc analysis of the bootstrapped deattenuated correlation between the two constructs [72,73]. Deattenuated correlations correct for measurement error by adjusting the observed correlation using Cronbach’s alpha for each scale. A bias-corrected and accelerated (BCa) bootstrap procedure with 1000 resamples was used to compute a 95% confidence interval for the deattenuated correlation [73]. Discriminant validity was supported if the upper bound of the confidence interval remained below 1.0, indicating that the constructs were not statistically indistinguishable [72].

## 3. Results

### 3.1. Participant Data

In total, 223 responses were collected. Of these, 22 were excluded for not meeting the previously described inclusion criteria. An additional 24 responses were removed due to missing data (MCAR assumption supported; see Methods), and 11 were excluded based on longstring response patterns. The final sample thus comprised N = 166 nursing trainees. Sample characteristics are summarized in Table 1.

The sample included trainees with diverse characteristics in terms of gender, age, year of training, and practical training setting. The distribution of these characteristics generally reflected the structure of the nursing trainee population in Germany [46], with younger trainees and those placed in hospital settings being somewhat overrepresented.

### 3.2. Descriptive Statistics

Table 2 displays the means, standard deviations, correlation matrix, Cronbach’s alphas, and McDonald’s omegas of the study variables.

All multi-item measures demonstrated acceptable internal consistency, with Cronbach’s alpha values ranging from 0.76 to 0.87 and McDonald’s omega values from 0.82 to 0.92; for both indices, values > 0.70 were considered acceptable [68].

As theoretically expected, ikigai showed a statistically significant—and according to conventional benchmarks [63]—strong positive correlation with work engagement (r = 0.50, *p* < 0.001). Personal resources (excluding ikigai) also correlated strongly positively with work engagement (r = 0.51, *p* < 0.001), and job resources showed a moderate positive correlation (r = 0.46, *p* < 0.001). In contrast, job demands were negatively associated with work engagement, showing a small but significant correlation (r = −0.16, *p* < 0.05).

### 3.3. Main Analysis

Table 3 presents the results of the hierarchical multiple linear regression examining the independent associations of ikigai, personal resources (excluding ikigai), job resources, and job demands with nursing trainees’ work engagement.

In Model 1, control variables (gender, age, practical training setting, and year of training), personal resources (excluding ikigai), job resources, and job demands were included. This model explained a substantial proportion of variance in work engagement (R^2^ = 0.370, adjusted R^2^ = 0.330, *p* < 0.001), corresponding to a large joint effect (f^2^ = 0.588) [63]. Personal resources (*p* < 0.001) and job resources (*p* < 0.001) showed statistically significant positive associations with work engagement, while job demands were not statistically significant (*p* = 0.532). None of the control variables were significantly associated with work engagement (all *p* > 0.05).

In Model 2, ikigai was added. The full model explained 40.3% of the variance in work engagement (R^2^ = 0.403, adjusted R^2^ = 0.361, *p* < 0.001), again indicating a large overall effect (f^2^ = 0.675). The inclusion of ikigai accounted for a statistically significant additional 3.3% of variance (ΔR^2^ = 0.033, *p* < 0.01), corresponding to a small incremental effect size according to Cohen’s standards [63] (f^2^ = 0.055).

A positive association between ikigai and work engagement was expected, and this was supported by the results. Ikigai was statistically significantly and positively associated with work engagement (β = 0.24, 95% CI [0.08, 0.41], *p* < 0.01). While the point estimate suggested a moderate effect, the confidence interval spanned from small to moderate values, warranting caution in interpreting the precise strength of the association.

Personal resources (excluding ikigai) were also positively associated with work engagement, as anticipated. The point estimate suggests a moderate effect, though the confidence interval includes values in the small range, indicating some uncertainty about the exact magnitude of the association (β = 0.25, 95% CI [0.08, 0.41], *p* < 0.01).

Job resources were significantly positively associated with work engagement, consistent with theoretical expectations. The estimated effect was moderate (β = 0.31, 95% CI [0.17, 0.45], *p* < 0.001), although some uncertainty extended into the small range.

Based on the job demands–resources theory [6] and prior studies [14,74], a weaker, and if present, negative association between job demands and work engagement was expected, given that the included factors represented hindrance demands (work overload, lack of formal rewards). The analysis revealed no statistically significant association (*p* = 0.850), which was consistent with expectations of a limited or absent effect.

As in Model 1, none of the control variables were statistically significant (all *p* > 0.05).

To further examine the distinctiveness of ikigai from the other personal resources, a post hoc analysis of the bias-corrected and accelerated (BCa) 95% confidence interval of the deattenuated correlation with the personal resources composite was conducted. The resulting 95% CI was [0.60, 0.87], which did not include 1.0 and thus supported discriminant validity.

## 4. Discussion

### 4.1. Theoretical Implications

Given the critical importance of work engagement in professional nursing—particularly in the context of global nursing shortages and growing demands for quality care—a substantial body of research has examined its antecedents [1,8]. Much of this work has been guided by the job demands–resources framework, which—being a theory of work design—emphasizes how organizational structures and job characteristics influence employee motivation and wellbeing [6]. Accordingly, prior research has primarily focused on job resources as antecedents of engagement. Although personal resources have also been investigated within this framework, this area has received comparatively less attention and has centered on a limited set of constructs such as self-efficacy, optimism, and self-esteem. Addressing this gap, our study investigated ikigai—a concept of life purpose—as a novel personal resource for work engagement in professional nursing practice.

Our findings offer several theoretical contributions. First, they provide empirical support for conceptualizing ikigai as a distinct personal resource relevant to work engagement. Consistent with the job demands-resources theory [6,7], ikigai demonstrated a statistically significant, positive association with work engagement in nursing trainees.

Our final model explained a substantial portion of variance in work engagement (R^2^ = 0.403, f^2^ = 0.675), with the addition of ikigai resulting in a statistically significant increase in explained variance (ΔR^2^ = 0.033, f^2^ = 0.055). This supports ikigai’s conceptual distinctiveness from established personal resources (self-efficacy, self-esteem, and optimism) [8,12,13,14] and recognized job resources [1,2,11]. While this incremental contribution may appear modest in absolute terms, it should be interpreted within the context of work engagement research, where numerous factors contribute to the outcome and our baseline model already explained substantial variance. This interpretation aligns with recent methodological perspectives that regard Cohen’s [63] conventional effect size guidelines as often arbitrary for complex psychological phenomena and note their tendency to overestimate empirical effect sizes in published research [75,76,77]. Notably, even if the effect size is small, the association affects a large number of nursing trainees [46], which underscores the significance of the results. Examining the standardized regression coefficients further illuminates ikigai’s role relative to other predictors. In our final model, ikigai demonstrated a significant positive unique association (β = 0.24) with work engagement, comparable to both other personal resources (β = 0.25) and job resources (β = 0.31). This pattern aligns with Mazzetti et al.’s meta-analysis [8], which found similar effect ranges for personal and job-related resources. A post hoc analysis of the deattenuated correlation between ikigai and other personal resources further supported their statistical distinctiveness, yielding a 95% BCa confidence interval of [0.60, 0.87], which excluded 1.0. Taken together, ikigai’s contribution represents a meaningful addition to our understanding of work engagement’s psychological antecedents, with potential practical significance for enhancing work engagement in nursing and functioning as a meaningful psychological resource alongside well-established antecedents of work engagement.

Second, our study contributes to the theoretical refinement of the job demands–resources framework by expanding the conceptual scope of personal resources. Existing research has predominantly focused on self-evaluative constructs (self-esteem), competence beliefs (self-efficacy), and outcome expectancies (optimism) [6,12,13]. These personal resources typically target specific aspects of self-perception or future anticipation. Our results suggest that existential constructs such as ikigai—which centers on meaning, purpose, and the significance of one’s existence [20,21,22,23,24]—also function as personal resources with distinct associations with work engagement. The existential orientation of ikigai provides a conceptual distinction from other personal resources that may explain its unique association with work engagement even after controlling for established factors. This distinction aligns with emerging research on religiosity and work engagement [78,79], where scholars have explored how faith-based meaning systems may function as personal resources. Our findings complement these efforts and collectively suggest the value of further investigating meaning-based constructs as potential personal resources within the job demands–resources framework, particularly in emotionally demanding professions like nursing [80] where questions of meaning and purpose are often salient.

Third, our study advances the field of ikigai research by extending it beyond its traditional domains. Previous research has primarily examined ikigai in relation to health-related outcomes, with a predominant focus on older populations and Japanese contexts [24,26]. While recent studies have validated the Ikigai-9 measurement instrument in Western countries such as Germany [24], the UK [26], and France [25], these and related efforts have largely maintained a focus on health aspects and outcomes (e.g., [33,81]) rather than exploring new domains of application. Our findings demonstrate that ikigai is not only measurable in Western contexts but also meaningfully related to work engagement among nursing trainees. This application to work-related outcomes suggests that ikigai represents a cross-culturally relevant construct with broader applicability than previously established. To the best of our knowledge, this study is among the first to empirically link ikigai with motivational outcomes in organizational settings, thereby extending ikigai research into organizational psychology broadly while specifically informing nursing management and education research.

### 4.2. Practical Implications

While future research is needed to assess the applicability of our findings across broader contexts, several practical implications already emerge for nursing management and education. Given that ikigai is considered a modifiable construct [33,81], healthcare organizations and nursing schools involved in training and instructing nursing trainees may implement evidence-based interventions to foster trainees’ sense of purpose and meaning, thereby supporting engagement. Although not specifically related to interventions to strengthen ikigai, meta-analyses of the efficacy of work engagement interventions have demonstrated that appropriate interventions are capable of strengthening personal resources [82,83].

First, interventions that strengthen social connectedness may promote ikigai. Prior research has shown that supportive relationships and social ties are a key foundation of life meaning [84,85] and that participating in interpersonal activities can increase ikigai [85,86]. Nursing schools and training providers can foster a “connection culture” [87] by promoting informal gatherings, team-based activities, shared break times, and physical work and learning environments conductive to interaction. Strengthening ties with peers, supervisors, and teaching staff can help create a relational climate that supports the development of ikigai. In addition, measures to optimize work–learning–life balance and develop a family-supportive organizational culture [87,88,89] could further enhance trainees’ ability to maintain meaningful personal relationships with friends, family, and significant others, which is another known contributor to ikigai [32,90,91].

In addition to social ties, it is known that personal goals, dreams, passions, and pursuit of deeply held values are constitutive for ikigai [21]. Thus, a second option to support the cultivation of nursing trainees’ ikigai could be to assist them to gain clarity about their future. Schippers and Ziegler [92] proposed a structured, evidence-based life crafting process grounded in positive psychology and the salutogenesis framework. This process involves identifying values and passions, reflecting on current and desired competencies and habits, envisioning one’s future career, and writing about the ideal and less ideal futures. It further includes setting specific goals, formulating “if–then” plans, and making public commitments to increase accountability. As artificial intelligence (AI) continues to advance rapidly and its further integration into nursing education and management is predicted [93], structured use of AI could also be an option worth exploring to support nursing trainees in finding their purpose [94]. Studies of the main use cases of AI suggest that AI is increasingly being used to determine and define one’s values, overcome blockages, and take steps towards self-development (e.g., advice on what to do next, reframing a problem, helping to focus) [95]. This would require a strategic framework that also addresses the associated challenges of transparency, ethical use, and AI literacy [93]. Interventions of this nature could be integrated into personal development programs or mentoring structures to support the development of purpose and, in turn, enhance work engagement through strengthened ikigai.

Third, promoting health-supportive behaviors may contribute to the development of ikigai and, consequently, work engagement. In prior research, lifestyle factors such as regular exercise, balanced nutrition, and adequate sleep have been linked to higher levels of ikigai [96,97,98]. Educational institutions and practice sites could foster such behaviors by nurturing a culture of health within an environment that prioritizes and promotes wellbeing at all levels [99,100]. Practical measures might include encouraging health-oriented leadership, providing access to exercise facilities, and offering healthy food choices in cafeterias and vending machines [100,101]. Additionally, training providers and schools may consider implementing recreation programs that align with Kono and Walker’s ikigai framework, which emphasizes experiences of enjoyment, effort, stimulation, and comfort as key to fostering a sense of purpose [20,98].

Finally, ikigai may serve as a practical indicator for early detection of disengagement risk. Given its links with both wellbeing and motivation, regular assessment using brief validated tools like the Ikigai-9 [23,24,25,26] could be integrated into trainee monitoring and support systems. This would allow early, targeted support for trainees at risk of low engagement or dropout.

### 4.3. Limitations and Research Directions

This study employed a cross-sectional design, limiting the ability to draw causal inferences [102,103,104,105]. While the findings demonstrate associations between variables, they do not establish directionality or causation. Future longitudinal studies and particularly intervention studies [6] are needed to investigate the causal pathways. In this context, finer-grained analyses of potential bidirectional relationships and gain cycles between ikigai and work engagement are warranted, as the job demands–resources theory conceptualizes work engagement to be both an outcome and a predictor of personal resources. This pattern is empirically supported with regard to self-efficacy, self-esteem and optimism [6,12,36], and potentially applicable to ikigai as well.

In addition, the school-mediated, geographically focused convenience sampling approach in this study introduced several limitations [106]. First, the non-probabilistic sampling limits external validity. Although the sample included nursing trainees from diverse backgrounds, younger trainees and those in hospital settings were somewhat overrepresented. Second, reliance on school cooperation for survey dissemination may have introduced gatekeeper bias [107], favoring participation from more engaged or better-resourced institutions. Third, there was a risk of self-selection bias, with participation potentially skewed toward trainees with higher engagement levels or greater interest in the study’s topic. Work and learning load may also have influenced who responded. Fourth, focusing recruitment on the federal state of Bavaria may have introduced regional bias. While vocational nursing training in Germany is regulated by a nationally standardized framework (the Nursing Professions Act), limiting structural variability across federal states, regional differences in healthcare and educational infrastructure, as well as socio-demographic and cultural factors, could influence how training is implemented and experienced and how constructs like ikigai are interpreted [108,109,110,111]. However, since Bavaria comprises metropolitan, urban, and rural areas with diverse healthcare institutions, nursing schools, population structures, and cultural value orientations, the internal heterogeneity of the sampled region may mitigate the risk of regional bias.

Moreover, because the total number of eligible individuals who received the survey invitation could not be determined, it was not possible to calculate a formal response rate. Although a low response rate does not necessarily imply substantial nonresponse bias [112], the inability to assess the response rate removes a commonly used indicator of such bias and limits evaluation of potential nonresponse bias. Future studies could address this by collaborating more directly with training institutions to document recruitment processes and enable assessment of sample coverage and potential nonresponse bias.

Finally, the sampling approach may have resulted in clustered data structures (e.g., nursing trainees nested within schools or training providers), which can violate the assumption of independent observations. Since the present analyses did not account for clustering, standard errors may be underestimated, increasing the risk of Type I error [113]. Future research should consider multilevel modeling to account for data dependencies, enable simultaneous analysis of individual- and group-level variables, and test whether associations differ across clusters [113].

Additionally, two of the scales measuring personal resources originally employed 5-point response formats and were linearly transformed to a 7-point scale to align with the format of other measures. While such rescaling preserves the relative spacing of response options and has been shown to produce comparable distributional characteristics such as means, variance, skewness, and kurtosis across 5- and 7-point formats [114], it may nonetheless influence psychometric properties such as internal consistency or construct validity. These potential effects should be considered when interpreting the findings.

Beyond addressing these limitations, several avenues for future research emerge. The present study focused on the direct association between ikigai and work engagement, in line with the direct effect model [12]. Future studies could expand this framework by examining potential mediators (e.g., work meaningfulness, positive emotions) and moderators (e.g., career stage, gender) to clarify the underlying mechanisms and conditions of this relationship. In addition, confirmatory factor analysis could be used to formally assess the discriminant validity of ikigai in relation to other personal resources [72].

Alternative theoretical models also warrant investigation [12]. For instance, the differential reactivity model suggests that ikigai may moderate the relationship between job resources and engagement, while the mediated effect model assumes that job resources and/or demands influence ikigai, which in turn affects work engagement.

Furthermore, while this study focused on trainees’ engagement during practical placements—which constitute the dominant portion of vocational training—future research should also examine the relationship between ikigai and engagement in theoretical instruction. In addition, it should be examined whether the findings can be generalized to university-based nursing training and to other professional groups, including nursing assistants, certified nursing professionals, and nursing managers. Finally, because this study was conducted in Germany, future studies should examine these associations in other national and cultural contexts to assess the broader applicability of the findings.

## 5. Conclusions

This study provides empirical support that ikigai—a sense of life purpose—is a distinct personal resource positively associated with work engagement among nursing trainees in Germany, even when controlling for established job resources, job demands, and other personal resources. These findings extend the job demands–resources theory by highlighting the motivational relevance of existential constructs in professional nursing.

Practically, the results suggest that fostering ikigai may represent a valuable complementary strategy to support trainee engagement. As ikigai is considered modifiable, healthcare organizations and educational institutions involved in training and instructing nursing trainees may implement interventions that strengthen purpose, including initiatives that promote social connection, structured reflection processes, mentoring, or nurturing a culture of health. Such approaches could enhance engagement and contribute to workforce retention in a time of critical nursing shortages.

## Figures and Tables

**Table 1:** Characteristics of the sample.

	%	n
**Gender**		
Female	77.1	128
Male	22.9	38
**Age (years)**		
≤18	23.5	39
19–20	37.3	62
21–24	18.1	30
≥25	21.1	35
**Current practical training setting**		
Hospital	59	98
Inpatient care facility	22.9	38
Outpatient care facility	15.1	25
Other	3	5
**Year of training**		
1st	32.5	54
2nd	32.5	54
3rd	35	58

Note: N = 166.

**Table 2:** Means, standard deviations, Pearson’s correlations, Cronbach’s alphas, and McDonald’s omegas of variables.

Variables	Items	Scale	*M*	*SD*	1	2	3	4	5	6	7	8	9	10	11	12
1. Ikigai	9	1–5	3.99	0.51												
2. Personal resources	16	1–7	5.41	0.73	0.62											
3. Job resources	12	1–6	4.14	0.75	0.29	0.32										
4. Job demands	7	1–6	3.61	0.95	−0.19	−0.04	−0.30									
5. Gender ^a^	1	-	0.23	0.42	0.03	0.03	0.13	−0.05								
6. Age (years)	1	-	22.70	7.40	0.19	0.22	−0.03	0.05	−0.02							
7. Setting: inpatient care facility ^a^	1	-	0.23	0.42	−0.05	0.03	−0.04	0.00	0.15	0.03						
8. Setting: outpatient care facility ^a^	1	-	0.15	0.36	0.01	0.00	0.07	−0.08	−0.03	0.01	−0.23					
9. Setting: other ^a^	1	-	0.03	0.17	0.09	0.04	−0.04	0.00	−0.01	0.17	−0.10	−0.07				
10. Year of training: 2nd ^a^	1	-	0.33	0.47	−0.13	−0.08	−0.01	0.15	0.11	−0.11	−0.07	0.14	0.03			
11. Year of training: 3rd ^a^	1	-	0.35	0.48	−0.02	−0.01	0.06	0.06	−0.07	0.00	−0.07	−0.20	−0.06	−0.51		
12. Work engagement	3	1–7	4.92	1.03	0.50	0.51	0.46	−0.16	0.08	0.15	0.00	0.03	0.02	−0.02	−0.06	
α					0.76	0.87	0.84	0.83	-	-	-	-	-	-	-	0.81
ω					0.82	0.90	0.89	0.92	-	-	-	-	-	-	-	0.83

Note: N = 166; *M* = mean; *SD* = standard deviation; α = Cronbach’s alpha; ω = McDonald’s omega; ^a^ dummy-coded variables: gender 1 = male, 0 = female; comparison category training setting: hospital; comparison category year of training: 1st; all |r| > 0.15 are significant at the level of 0.05, all |r| > 0.19 are significant at the level of 0.01.

**Table 3:** Hierarchical multiple linear regression predicting work engagement.

	Model 1					Model 2				
	B	SE	β	95% CI	*p*	B	SE	β	95% CI	*p*
**Control variables**										
Gender ^a^	0.05	0.16	0.02	[−0.11, 0.15]	0.748	0.04	0.16	0.02	[−0.11, 0.14]	0.789
Age (years)	0.01	0.01	0.07	[−0.06, 0.21]	0.267	0.01	0.01	0.06	[−0.07, 0.19]	0.348
Setting: inpatient care facility ^a^	−0.05	0.17	−0.02	[−0.15, 0.12]	0.785	0.00	0.16	0.00	[−0.13, 0.13]	0.989
Setting: outpatient care facility ^a^	−0.04	0.20	−0.01	[−0.15, 0.12]	0.832	−0.02	0.19	−0.01	[−0.14, 0.12]	0.899
Setting: other ^a^	0.00	0.39	0.00	[−0.13, 0.13]	0.992	−0.06	0.39	−0.01	[−0.14, 0.12]	0.878
Year of training: 2nd ^a^	−0.04	0.17	−0.02	[−0.17, 0.14]	0.823	0.01	0.17	0.01	[−0.15, 0.16]	0.934
Year of training: 3rd ^a^	−0.19	0.17	−0.09	[−0.24, 0.07]	0.262	−0.16	0.16	−0.07	[−0.22, 0.08]	0.344
**Independent variables**										
Personal resources	0.54	0.10	0.39	[0.25, 0.52]	<0.001	0.35	0.12	0.25	[0.08, 0.41]	0.004
Job resources	0.45	0.10	0.32	[0.18, 0.47]	<0.001	0.42	0.10	0.31	[0.17, 0.45]	<0.001
Job demands	−0.05	0.08	−0.04	[−0.18, 0.09]	0.532	−0.01	0.07	−0.01	[−0.15, 0.12]	0.850
**Additional predictor (Model 2)**										
Ikigai	-					0.48	0.17	0.24	[0.08, 0.41]	0.004
**Model statistics**										
R^2^	0.370					0.403				
Adjusted R^2^	0.330					0.361				
F (df)	9.11 (10,155)					9.46 (11,154)				
*p*	<0.001					<0.001				
∆R^2^	-					0.033				
F for ∆R^2^ (df)	-					8.52 (1,154)				
p for ∆R^2^	-					0.004				

Note: N = 166. B = unstandardized regression coefficients, SE = standard errors of B, ß = standardized coefficients, 95% CI = 95% confidence intervals for β; ^a^ dummy-coded variables: gender 1 = male, 0 = female; comparison category setting: hospital; comparison category year of training: 1st.

## Data Availability

The data presented in this study are available on request from the corresponding author.

## Public Involvement Statement

Nursing professionals, including certified nurses and nurse educators, were involved in the development of the study materials. They participated in a pre-test of the questionnaire and provided feedback on item clarity, structure, and usability. Their input was used to refine the final version of the instrument. No further public involvement occurred in the design, conduct, reporting, or dissemination of this research.

## Guidelines and Standards Statement

This manuscript was drafted against the STROBE Checklist for cohort, case-control, and cross-sectional studies [115].
